# Little heterosis found in diploid hybrid potato: The genetic underpinnings of a new hybrid crop

**DOI:** 10.1093/g3journal/jkac076

**Published:** 2022-04-23

**Authors:** James R Adams, Michiel E de Vries, Chaozhi Zheng, Fred A van Eeuwijk

**Affiliations:** 1 Biometris, Mathematical and Statistical Methods, Wageningen University and Research, 6700 HB Wageningen, The Netherlands; 2 Solynta, 6703 HA Wageningen, The Netherlands

**Keywords:** diploid hybrid potato, GCA, SCA, sparse crossing design, heterosis, MPP, Multiparental Populations, Multiparent Advanced Generation Inter-Cross (MAGIC)

## Abstract

Hybrid potato breeding has become a novel alternative to conventional potato breeding allowing breeders to overcome intractable barriers (e.g. tetrasomic inheritance, masked deleterious alleles, obligate clonal propagation) with the benefit of seed-based propagule, flexible population design, and the potential of hybrid vigor. Until now, however, no formal inquiry has adequately examined the relevant genetic components for complex traits in hybrid potato populations. In this present study, we use a 2-step multivariate modeling approach to estimate the variance components to assess the magnitude of the general and specific combining abilities in diploid hybrid potato. Specific combining ability effects were identified for all yield components studied here warranting evidence of nonadditive genetic effects in hybrid potato yield. However, the estimated general combining ability effects were on average 2 times larger than their respective specific combining ability quantile across all yield phenotypes. Tuber number general combining abilities and specific combining abilities were found to be highly correlated with total yield’s genetic components. Tuber volume was shown to have the largest proportion of additive and nonadditive genetic variation suggesting under-selection of this phenotype in this population. The prominence of additive effects found for all traits presents evidence that the mid-parent value alone is useful for hybrid potato evaluation. Heterotic vigor stands to be useful in bolstering simpler traits but this will be dependent on target phenotypes and market requirements. This study represents the first diallel analysis of its kind in diploid potato using material derived from a commercial hybrid breeding program.

**Key message**

Hybrid vigor was detected for multiple traits in diploid hybrid potato. Additive gene action was most prominent in tuber yield and should be the primary target within hybrid breeding programs.

## Introduction

Potato (*Solanum tuberosum*), a plant species once isolated to the continents of southern and central America, is now a crop that spans over 17 million hectares of crop-land worldwide (FAOSTA 2021). It is the most prominent of noncereals and is considered by many a major keystone in guaranteeing food security for both local and global communities. Prized for their edible starch-rich tubers, potato meets the demand of several key industries including the fresh, processing, and seed potato markets with a global gross value of 140.5 billion USD as of 2019. As a field crop, potato has a competitive harvest index of 0.85 (in contrast to 0.4–0.6 seen in other crops) in conjunction with a high water productivity (Hay 1995; Lutaladio and Castaldi 2009). There is also ample variation in potato’s tuberization timing requiring as few as 75 days from planting to harvest. All the above make potato a highly productive crop amenable to a variety of cropping systems capable of supplying valuable starch with less agronomic input.

Despite potato’s growing economic and societal importance, rates of crop improvement in complex traits have not kept in step with other major crops over the past century (Douches *et al.* 1996; Hirsch *et al.* 2013). Reasons for these deficits in genetic gain are numerous (e.g. market segmentation, large inventory of quality traits, etc.) but many of them stem from the complexities of potato’s evolution and domestication. Potato’s tetraploidy is an oft-cited stumbling block for breeders impeding the ability to fix beneficial loci, and conversely, remove deleterious sites harbored across the genome (Lian *et al.* 2019; Zhang *et al.* 2019). Not only does polyploidy mask deleterious loci from traditional forces of selection but it also impacts the length of time for site fixation even under genetic drift leading to greater maintenance of heterozygosity over time (Bartlett and Haldane 1934). Taken together with a very strong self-incompatibly mechanism, potato could best be described as a fortified heterozygous out-crosser. These biological realities shaped potato breeding from the beginning with breeders conducting crosses between promising heterozygous individuals followed by the evaluation of large nurseries in search of decent complementation (Simmonds 1979). These F1 nurseries were then subjected to as many as 8 subsequent rounds of clonal selection until only elite candidates were left (Bradshaw 2017). While in some ways, this method of clonal breeding is quite efficient (all genetic factors are effectively *fixed* at the creation of the F1), it is widely known for being a long process from generation of the nursery to variety release. Because the success of clonal breeding is highly dependent on the generation of enough novel genotypes in the F1, it takes as many as 9 years to sufficiently bulk tubers in conjunction with applying appropriate selection pressure (Bryan 1981; Tai and Young 1984). It should be noted that while there have been proposals to optimize conventional clonal breeding (Neele *et al.* 1991; Bradshaw *et al.* 2003), many of the aforementioned issues are simply implicit to breeding tetraploid potato.

One solution to this comprehensive set of challenges is the adaptation of potato from a tetraploid clonal crop to that of a diploid inbred–hybrid one, an idea that has existed in some form for over 60 years (Hougas and Peloquin 1958). The benefits of such a change, if possible, are manifold; Diploids only take one generation to half their heterozygosity in contrast to an autotetraploid which takes upwards of 4 generations making the production of pure-breeding lines plausible in the former. As an extension of this, superior genetic performance in the final marketed variety is not dependent on a single crossing event that generated the original F1 (as it is in conventional clonal breeding) but is accomplished through multiple stages (e.g. parental pool improvement, parental line development, hybrid crossing, etc.). This is not to mention other logistical niceties such as the ease of producing and storing true potato seed over vegetative propagule (Cock 1983; Pallais 1991; Thomas-Sharma *et al.* 2016). Despite the potential of diploid potato, however, it was not until the cusp of the 21st century that it became broadly feasible. Many picked up on the work of Hosaka and Hanneman (1998) and began the process of generating self-compatible populations through the use of *Sli*. Lindhout *et al.* (2011) were one of the first to confirm the commercial viability generating diploid potato populations capable of inbreeding using an *Sli* donor. Several subsequent studies not only corroborated that inbred populations in diploid potato were possible (Alsahlany *et al.* 2021), but hybrids generated from these populations resulted in a crop that could compete in the same space as tetraploid potato (Stockem *et al.* 2020; Zhang *et al.* 2021). While diploid hybrid potato (DHP) populations are now extant across the world, there is at this time little known about the genetic components controlling complex traits as DHP is still a young hybrid crop. Understanding this is an imperative for potato breeders in order to structure breeding programs that are able to best exploit the genetic variation available to DHP.

We set out to inspect tuber yield in a large DHP test cross. To do this, we performed a joint evaluation of total yield (TY) along with 2 of its simpler yield components, average tuber volume and total tuber number (TN), and partitioned their underlying genetic effects into additive and nonadditive components. This trait and genetic decomposition was done to inspect 2 broad questions: (1) Which tuber phenotypes in this population were responsible for the variation seen in TY and (2) are these yield phenotypes primarily under the control of additive or nonadditive gene action? This latter question holds particular weight as it gives insight on where the focal point of DHP breeding should lie. We put forward a 2-part modeling approach to utilize intrablock information to estimate the general and specific combining abilities of our hybrid parents and crosses, respectively. Our study presents the first diallel study in DHP using highly inbred parents derived from a commercial breeding program.

## Materials and methods

### Crosses and trials

A panel of 400 inbred parents was selected and crossed according to distinct selection criteria related to fertility and agronomic traits yielding 806 successful F1 crosses. These parents were produced from an experimental population derived from several backgrounds including *tuberosum* and several wild species (e.g. *Solanum chacoense*; Lindhout *et al.* 2018). In the Spring of 2019, all hybrid true potato seed (TPS) were sown in trays and grown out in a greenhouse. In May, all seedlings were transplanted at stage 105 development (see Kacheyo *et al.* 2022) into 2 field trials located in the Dutch towns of Est and Heelsum. Both trials utilized a double ridge design with 8 plants per ridge with a total of 16 plants per plot; this design was chosen to minimize within-plot variation while reducing planting costs across each trial (Stockem *et al.* 2022). Plots were organized in an augmented randomized complete block design with 2 blocks and 3 internal controls used across each block. All 806 F1 hybrids were planted in Heelsum with a subset of 608 hybrids planted in Est. Trial conditions were similar with regard to field management and scoring. One distinguishing factor between trials was their soil conditions with Est being characterized by a light clay composition and Heelsum conversely by distinctively sandy conditions (see Table 1). Both trials were conducted through the summer until haulm killing in early September followed by subsequent harvest 2 weeks later. All hybrids were scored by several criteria including relevant yield-related traits which are our primary focus for this study, i.e. TY, TN, and TV. Total TN was measured as the total number of tubers harvested from a given plot of 16 plants. Tuber volume (TV) was calculated using an average over all tubers harvested per plot using a tuber’s length, width, and depth dimensions to calculate volume using an ellipsoid approximation. Lastly, TY was calculated through a transformation of the total tuber weight of a plot to estimate the approximate yield in units of Mg Ha^−1^. These traits were collected for all tubers above 20 mm in length via an automated pipeline described in Stockem *et al.* (2020).

**Table 1.** Agronomic properties of the screening trials conducted in Est and Heelsum.

					Soil composition^*a*^			
Locations	Year	Rows	Columns	Hybrids	Sand	Silt	Clay	Organic matter
Est	2019	20	65	608	23	45	28	3
Heelsum	2019	20	85	806	76	16	2	6

a Soil characteristics presented as percentage.

### Spatial models

**Fig. 1.**
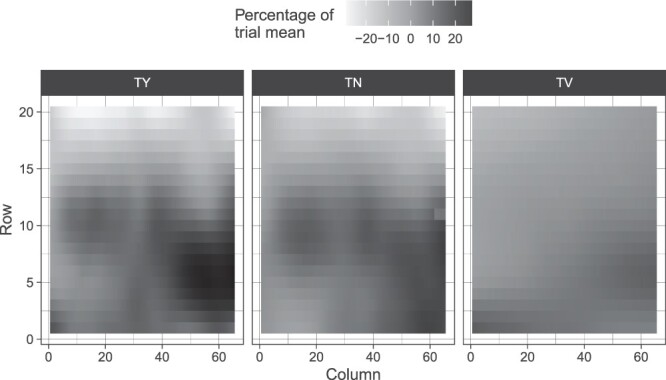
Estimated spatial trends scaled by trial mean for TY (Mg Ha^−1^), TN (number of tubers per plot), and TV (average cm^3^ per plot) within the Est screening trial.

**Fig. 2.**
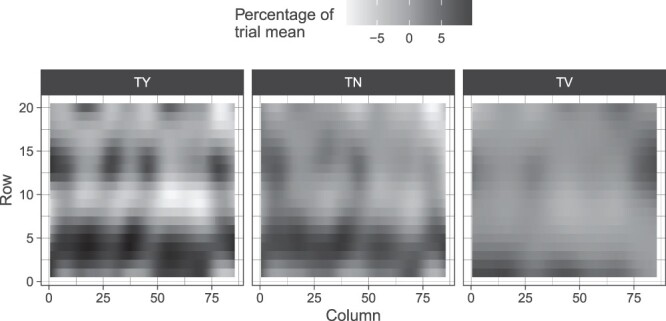
Estimated spatial trends scaled by their trial mean for TY (Mg Ha^−1^), TN (number of tubers per plot), and TV (average cm^3^ per plot) within the Heelsum screening trial.

This present study used a 2-step modeling approach where each field trial was modeled separately accounting for factors like field design, control effects, and spatial heterogeneity allowing for the extraction of spatial trends and detrended phenotype data. This was followed by modeling of genetic components simultaneously for all phenotypes. The first step was accomplished by partitioning field effects into local and global trends using 2D penalized splines. This was performed using the Spatial Analysis of Field Trials with Splines (SpATS) library available through the comprehensive R archive network (CRAN; Rodríguez-Álvarez *et al.* 2018). While many attested methods capable of handling geospatial trends exist, the spatial smoothing approach offered through SpATS was chosen for a few reasons. Often, genetic modeling requires the creation of many spatial models with different spatial structures in order to identify the most satisfactory spatial model. SpATS, conversely, does not follow this procedure and is capable of offering comparable genotype estimates with the best traditional spatial model (Velazco *et al.* 2017). Along with this, SpATS provides a number of internal methods allowing for intelligible and simple model diagnostics to help elucidate the predominant factors for a given field trial. We chose to model field dimensions using SpATS’ PS-ANOVA method which is capable of taking the bivariate surface and decomposing it into multiple spatial components all defined by one smoothing parameter (Lee *et al.* 2013). The resultant model equation is then:

(1) $$y_{\mathrm{chmn}}=\beta_{c}+g_{h}+r_{m}+c_{n}+f_{ps}(m,n)+\varepsilon_{\mathrm{chmn}},$$

where *β_c_* is a fixed effect for whether the hybrid was a control variety, *g_h_* is a random effect for hybrid, *h*, *r*, and *c* are random effects for row, *m*, and column, *n*. The row and column coordinates were also used by the 2D penalized-spline function, *f_ps_*. The PS-ANOVA was parametrized using 19 and 83 internal knots for Heelsum and 19 and 63 internal knots for Est. The large number of internal knots resulted in longer computational time, but was selected to mirror the number of plots along each row and column for each trial. Third-degree polynomial B-splines with second-degree penalties were used for all spatial models, from which, spatial trends were derived and then subsequently used to detrend the phenotype data for each trait:

(2) $$y_{hk}^{*}=y_{\mathrm{hmn}}-\left(r_{m}+c_{n}+f_{ps}(m,n)\right),$$

where $y^{*}$ represents the corrected phenotype with systematic spatial trends removed. Each spatial trend was presented as a percentage deviation from the trial mean (see Figs. 1 and 2). Along with this, every spatial model was evaluated on the basis of effective and nominal dimension number estimated for each model effect. These were used to evaluate the number of parameters estimated for smoothing and random terms (see Supplementary Tables 1 and 2). Taking the ratio between effective and nominal dimensions for random hybrid effects has the benefit of being interpreted as a generalized heritability where the effective dimension number for a hybrid genotype effect (the trace of its hat matrix) is divided by its nominal dimension (the rank of its design matrix) allowing for a direct assessment of genetic variation exhibited within a field trial (Oakey *et al.* 2006).

### Genetic models

**Fig. 3.**
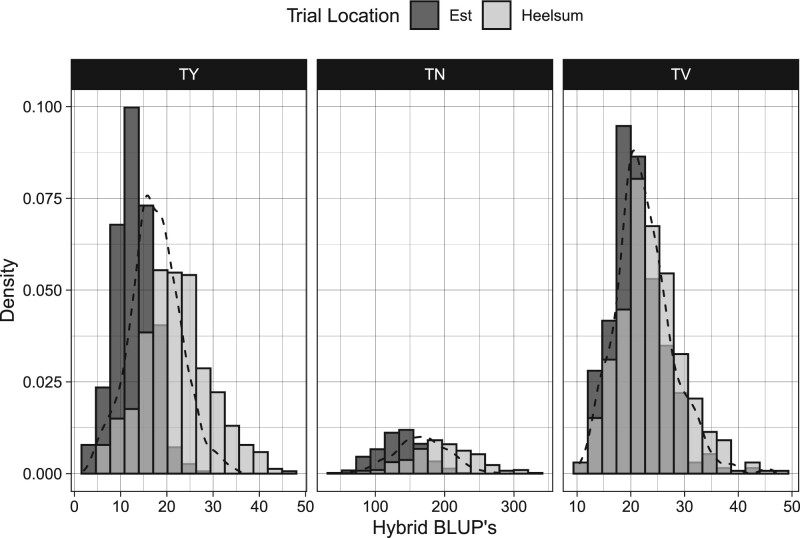
Hybrid BLUPs for TY (Mg Ha^−1^), TN (number of tubers per plot), and TV (average cm^3^ per plot) conditioned on Est and Heelsum trials. Across-location BLUPs visualized through black density curve.

For genetic modeling, F1 hybrids were included in the subsequent analysis based upon 2 criteria: their presence in both screening trials, and whether both parents of a hybrid were utilized in at least 2 crosses. The former criteria were to ensure estimation of each genotype location combination while the latter was to exclude unconnected crossing sets to guarantee demarcation of parental and cross-wise effects. This resulted in the selection of 225 parental lines which gave rise to 495 F1 hybrid progeny. This panel of hybrids was first utilized in the following multitrait multilocation model:

(3) $$y_{\mathrm{fgk}}^{*}=\mu +\beta_{f}+h_{g}+t_{fg}+\varepsilon_{\mathrm{fgk}},$$

with *μ* being the global mean, and *β_f_* the field effect for trial location, *f*. *h_g_* is the random hybrid effect for hybrid, *g*, while *t_fg_* is the random hybrid by trial interaction for hybrid, *g*, and trial, *f*, and $\varepsilon_{\mathrm{fgk}}$ is the residual for hybrid, *g*, field trial, *f*, and replicate, *k*. From this hybrid model, best linear unbiased predictions (BLUPs) were made for each phenotype and hybrid over all trials ($E[y_{g}|h]$) as well as conditioned on each trial ($E[y_{fg}|h,t]$; see Fig. 3); variance components were also extracted from this hybrid model for all 3 tuber phenotypes (Supplementary Table 3).

The intent of our paper is not merely to retrieve hybrid estimates, but further decompose these estimates into distinct additive and nonadditive components. In the context of plant populations, this is traditionally done through a series of controlled crosses between a set of parents which allows for the separation of the parental mean, or the general combining ability (GCA), and the deviation from the expected mean of a cross, or the specific combining ability (SCA; Sprague and Tatum 1942). These 2 parameters also have the benefit of being interpreted in terms of the genetic variances of a population. The variance attributable to GCA is equal to the covariance between half siblings while the variance attributed to SCA is equal to the covariance between full siblings subtracted by twice covariance of half siblings (Bernardo 2002). Such models have been made for a variety of population designs (full diallel, half diallel, factorial, etc.) with different effect structures depending on the intent of inquiry. For example, genetic effects can be modeled as fixed if the interest is to provide valid performance estimates for a given cross or they can be treated as random if the variance of effect sampled from a population is desired to be studied (Eisenhart 1947). Additionally, these models can be expanded or simplified accommodating reciprocal effects, trial location or environment interactions, population structures, and so forth. For our purposes, we extend Griffing’s model II (Griffing 1956) into a multivariate context where hybrid yield can be described as following:

(4) $$y_{\mathrm{fijk}}^{*}=\mu +\beta_{f}+g_{i}+g_{j}+t_{fi}+t_{fj}+\varepsilon_{\mathrm{fijk}},$$

where, identical to equation (3), *μ* and *β_f_* correspond to a global mean and field trial effect, respectively, for field trial, *f*. *g_i_* and *g_j_* are random effects for the GCAs of parents’ *i* and *j*, respectively, with *t_fi_* and *t_fj_* being their respective field trial and parental interactions for trial location, *f*. $\varepsilon_{\mathrm{fijk}}$ is the model’s residual for replicate, *k*, on progeny of parents’, *i* and *j*, evaluated in trial, *f*. This model, containing only the additive genetic effects, will hereon be denoted as *M*_0_.

This model can be expanded further to include hybrid cross-wise effects with the addition of the SCA and SCA by environment interaction. This final model then has the form:

(5) $$y_{\mathrm{fijk}}^{*}=\mu +\beta_{f}+g_{i}+g_{j}+t_{fi}+t_{fj}+s_{ij}+r_{\mathrm{fij}}+\varepsilon_{\mathrm{fijk}},$$

where *s_ij_* is the SCA for parents’ *i* and *j* with *r_fij_* being their respective interaction with trial location, *f*. With the addition of these 2 random effects, the model will be denoted as *M_f_* going forward.

Random effects for all genetic models were assumed to proceed from a multivariate normal distribution centered about zero with an unstructured covariance matrix applied over the 3 tuber phenotypes in the form of:

(6) $$u∼\mathrm{MVN}(0,\Sigma_{u}⊗I)\;\;\;\;\Sigma_{u}=\left[\begin{array}{ccc} \sigma_{u_{\mathrm{TY}}}^{2} & \sigma_{u_{\mathrm{TY},\mathrm{TN}}} & \sigma_{u_{\mathrm{TY},\mathrm{TV}}}\\ \sigma_{u_{\mathrm{TN},\mathrm{TY}}} & \sigma_{u_{\mathrm{TN}}}^{2} & \sigma_{u_{\mathrm{TN},\mathrm{TV}}}\\ \sigma_{u_{\mathrm{TV},\mathrm{TY}}} & \sigma_{u_{\mathrm{TV},\mathrm{TN}}} & \sigma_{u_{\mathrm{TV}}}^{2} \end{array}\right]$$

for any given random effect, *u*. This includes the hybrid ($\Sigma_{h}$) and hybrid trial location interaction ($\Sigma_{\mathrm{hxe}}$) from model (3), the GCA ($\Sigma_{\mathrm{gca}}$) and GCA by trial location interaction ($\Sigma_{\mathrm{gxe}}$) in models’ (4) and (5), and the SCA ($\Sigma_{\mathrm{sca}}$) and SCA by trial location interaction ($\Sigma_{\mathrm{sxe}}$) in model (5). This also applies for the residual ($\Sigma_{\varepsilon }$) in all genetic models listed here.

### Variance ratios and genetic correlations

**Fig. 4.**
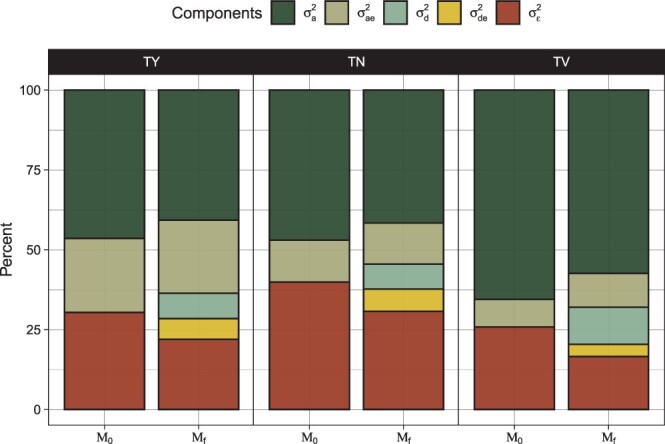
Partitioning of total phenotypic variance into additive ($\sigma_{a}^{2}$), dominant ($\sigma_{d}^{2}$), interactive ($\sigma_{ae}^{2}$ & $\sigma_{de}^{2}$), and residual ($\sigma_{\varepsilon }^{2}$) genetic components across each genetic model (*M*_0_ and *M_f_*) for TY, TN, and average TV.

To study all relevant effects, variance components were estimated and extracted from models *M*_0_ and *M_f_*. These variance components were used for 2 general purposes: (1) to derive ratios of effects within traits and (2) to produce genetic correlations between traits and trial locations. These components were first used to derive several important genetic parameters including variation due to additive genetic effects ($2\cdot \mathrm{Diag}(\Sigma_{\mathrm{gca}})={\left(\sigma_{a_{\mathrm{TY}}}^{2},\sigma_{a_{\mathrm{TN}}}^{2},\sigma_{a_{\mathrm{TV}}}^{2}\right)}^{T}$), variation due to dominance ($\mathrm{Diag}(\Sigma_{\mathrm{sca}})={\left(\sigma_{d_{\mathrm{TY}}}^{2},\sigma_{d_{\mathrm{TN}}}^{2},\sigma_{d_{\mathrm{TV}}}^{2}\right)}^{T}$), and their respective environmental interactions ($2\cdot \mathrm{Diag}(\Sigma_{\mathrm{gxe}})={\left(\sigma_{ae_{\mathrm{TY}}}^{2},\sigma_{ae_{\mathrm{TN}}}^{2},\sigma_{ae_{\mathrm{TV}}}^{2}\right)}^{T}$ and $\mathrm{Diag}(\Sigma_{\mathrm{sxe}})={\left(\sigma_{de_{\mathrm{TY}}}^{2},\sigma_{de_{\mathrm{TN}}}^{2},\sigma_{de_{\mathrm{TV}}}^{2}\right)}^{T}$). Proportion of total phenotypic variance was then examined with respect to these genetic variances along with each trait’s residual variance (Fig. 4). These genetic parameters were then used to calculate several variance ratios (see Table 2) including broad and narrow-sense heritabilities, *H*^2^ ($\frac{\sigma_{a}^{2}+\sigma_{d}^{2}}{\sigma_{p}^{2}}$) and *h*^2^ ($\frac{\sigma_{a}^{2}}{\sigma_{p}^{2}}$), respectively, dominance ratios ($d^{2}=\frac{\sigma_{d}^{2}}{\sigma_{p}^{2}}$), additive portion of genetic variation ($\frac{\sigma_{a}^{2}}{\sigma_{G}^{2}}$), and additive by trial location portion of total genetic by environment variation ($\frac{\sigma_{ae}^{2}}{\sigma_{GE}^{2}}$). Total phenotypic variation ($\sigma_{p}^{2}$) was computed by scaling $\sigma_{ae}^{2}$ and $\sigma_{de}^{2}$ by the total number of field trials and $\sigma_{\varepsilon }^{2}$ by the product of the number of field trials and the number of replicates used within each trial, i.e. $\sigma_{p}^{2}=\sigma_{a}^{2}+\frac{\sigma_{ae}^{2}}{2}+\sigma_{d}^{2}+\frac{\sigma_{de}^{2}}{2}+\frac{\sigma_{\varepsilon }^{2}}{4}$.

**Table 2.** The broad and narrow-sense heritabilities (*H*^2^ and *h*^2^, respectively), dominance ratio (*d*^2^), proportion of additive genetic variation ($\frac{\sigma_{a}^{2}}{\sigma_{G}^{2}}$), and proportion of additive genotype by environment interaction over total genotype by environment interaction ($\frac{\sigma_{ae}^{2}}{\sigma_{GE}^{2}}$) estimated from the *M_f_* model for TY, TN, and average TV.

	TY	TN	TV
*H* ^2^	0.71	0.74	0.86
*h* ^2^	0.59	0.62	0.71
*d* ^2^	0.12	0.12	0.14
$\frac{\sigma_{a}^{2}}{\sigma_{G}^{2}}$	0.84	0.84	0.83
$\frac{\sigma_{ae}^{2}}{\sigma_{GE}^{2}}$	0.78	0.65	0.74

**Fig. 5.**
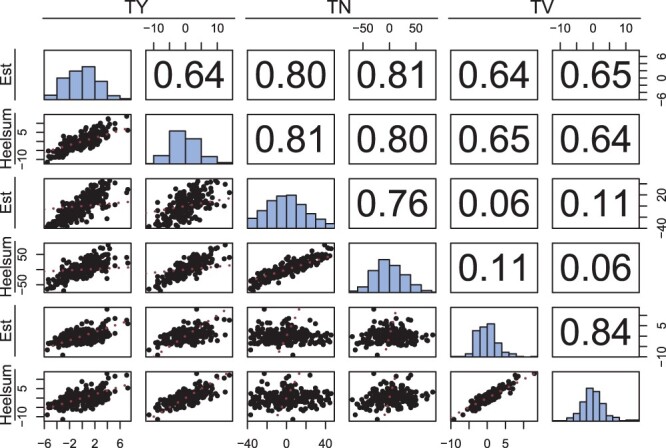
GCA pairwise comparisons with scatter plot (lower triangle), genetic correlations derived from *M_f_*’s variance components (upper triangle), and marginal distributions (the diagonal) of BLUPs for TY (Mg Ha^−1^), TN (total number of tubers per plot), and average TV (cm^3^) in Est and Heelsum. The identity is provided in red for each scatter plot (lower triangle).

**Fig. 6.**
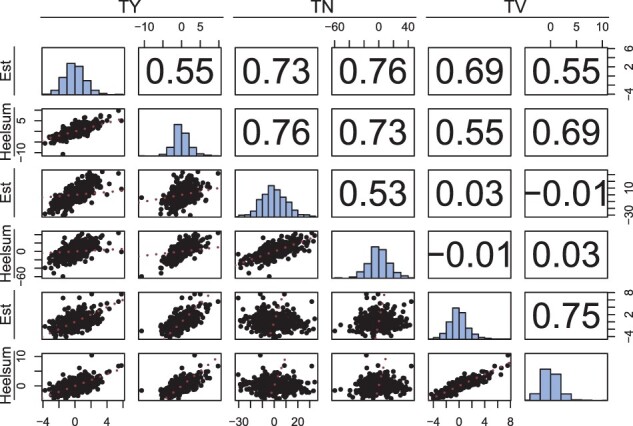
SCA pairwise comparisons with scatter plot (lower triangle), genetic correlations derived from *M_f_*’s variance components (upper triangle), and marginal distributions (the diagonal) of BLUPs for TY (Mg Ha^−1^), TN (total number of tubers per plot), and average TV (cm^3^) SCA in Est and Heelsum. The identity is provided in red for each scatter plot (lower triangle).

Second, variance components from model *M_f_* were used to estimate genetic correlations in GCA and SCA effects. These include genetic correlations between traits (e.g. $\frac{cov(gca_{TY},gca_{TV})}{\sigma_{TY}\sigma_{TV}}$), intraclass correlation coefficients between trial locations (e.g. $\frac{\sigma_{gca}^{2}}{\sigma_{gca}^{2}+\sigma_{gxe}^{2}}$), and genetic correlations between traits and trial locations (e.g. $\frac{cov(gca_{TY},gca_{TV})+\mathrm{cov}(gxe_{TY},gxe_{TV})}{\sqrt{\left(\sigma_{gca_{TY}}^{2}+\sigma_{gxe_{TY}}^{2})\cdot (\sigma_{gca_{TV}}^{2}+\sigma_{gxe_{TV}}^{2}\right)}}$). These were computed for the GCA and GCA by trial location effects (Fig. 5) as well as SCA and SCA by trial location effects (Fig. 6). Each of these is presented with multivariate scatter plots and marginal BLUP distributions for all effects.

### Hypothesis testing

To evaluate statistical evidence of heterosis (through SCA term), we perform a hypothesis testing procedure on the original *M*_0_ [equation (4)] and *M_f_* [equation (5)] models as well as on the univariate analogs for each trait. This was done to assess the meaningful addition of SCA effects with respect to each phenotype without consideration of extra covariance parameters in *M*_0_ and *M_f_*. *M*_0_ then, along with its univariate analogs, represents a null model where the additional effects from *M_f_*, are constrained to zero. Therefore, we can construct a nonstandard hypothesis test where:

(7) $$\begin{array}{c} H_{0}:\sigma_{\mathrm{sca}}^{2},\sigma_{\mathrm{sxe}}^{2}=0\\ H_{1}:\sigma_{\mathrm{sca}}^{2},\sigma_{\mathrm{sxe}}^{2}>0 \end{array},$$

which can be evaluated directly through the following likelihood ratio test where:

(8) $$\Lambda (y)=-2ln\left(\frac{\ell (M_{0})}{\ell (M_{f})}\right)=2(\mathrm{loglik}(M_{f})-\mathrm{loglik}(M_{0})).$$

This test is nonstandard because it follows a special case where testing is occurring on the boundary of the parameter space which is often taken into account using a mixed $\chi^{2}$ distribution following (Self and Liang 1987). For our testing purposes, we used a $0.25\chi_{0}^{2}$ + $0.50\chi_{1}^{2}$ + $0.25\chi_{2}^{2}$ mixture distribution. These tests are also accompanied with the Akaike information criterion (AIC) for each univariate and multivariate set of models (Table 3). Under *H*_1_, the hybrid genetic effect from our original model (3) should equal the sum of the GCA and SCA effects specified in model (5). Our modeling procedure for all multivariate models began with the estimation of variance components and production of genetic correlations on BLUPs in the univariate analogs (i.e. *M*_0_ and *M_f_*), which were used to initialize the unstructured covariance matrices for all random effects in model’s *M*_0_ and *M_f_*. All models were fitted through restricted (residual) maximum likelihood (REML) using ASReml-R 4 (Butler *et al.* 2017). REML-based procedures have come into popular usage over the past 2 decades due to their ability to provide estimators both consistent and asymptotically normal even under conditions with nonorthogonal sets of random predictors which are particularly useful while using sparse crossing designs (Searle *et al.* 1992). This is not to mention the volume of diallel-based literature where REML is the invoked method of choice for reasons which will not be discussed here [Möhring *et al.* (2011) provide an excellent review on the topic]. Because the underlying crossing sets are sparse, identifiability of model’s *M*_0_ and *M_f_* was tested following (Xenakis 2019) to ensure that statistically valid estimates could be derived from all genetic models.

**Table 3.** Likelihood ratio tests for *M*_0_ and *M_f_* genetic models together with each model’s AIC for the Multitrait model (MT), TY TN, and average TV.

	AIC	Λ(y)	P(Λ(y)) ^a^
MT			
*M*_0_	26,665	586	***
*M_f_*	26,103		
TY			
*M*_0_	7,992	132	***
*M_f_*	7,864		
TN			
*M*_0_	15,778	92	***
*M_f_*	15,690		
TV			
*M*_0_	7,008	235	***
*M_f_*	6,778		

*** ^a^ P(Λ(y))<0.001 .

## Results

### Spatial components

Spatial models for the Est and Heelsum trials were estimated for each yield component. All spatial models successfully converged with credible spatial trends for both trial locations. Model residuals for all environmental models showed little to no evidence of deviation from normality; the following suggests successful delineation of spatial components for all traits in both field trials.

TY and TN exhibited evidence for strong local trends with a row effect contributing to the spatial trend in Est (Fig. 1). While these same components also impacted TV, it was not nearly so prominent (see Supplementary Data 1). The similar magnitude of row effects on TN and TY can also be observed through the ratio of effective and nominal dimensions which were identical for these 2 traits (0.68) in contrast to TV (0.47). Additionally, the effective nominal dimension ratio for hybrids (i.e. a generalized heritability) was highest in TV followed by TY and lastly TN (see Supplementary Table 1).

Overall, the spatial trends in the Heelsum trial were less severe than those evaluated in the screening trial in Est. Interquartile ranges of field effects were between −8.96% and 9.04% of the trial mean for TY in Est while the range was −2.85% and 3.55% of the trial mean for yield in Heelsum (Fig. 2). The differences in the magnitude of spatial components between these 2 trials were also similar for TN and TV. Nonetheless, the estimated spatial components did have a modest effect on all yield-related components in Heelsum. In particular, random effects for the field column had a minor impact on TV (0.55), TN (0.48), and TY (0.45) (see Supplementary Table 2). General heritability estimates for Heelsum were akin to those observed in Est with values of 0.9, 0.82, and 0.88 for TV, TN, and TY, respectively.

### Hybrid estimates

Using spatially corrected phenotypes from model (1), we extracted both marginal and conditioned hybrid BLUPs from model (3) (Fig. 3). Generally, hybrid performance was far greater in Heelsum over Est. The trial mean for TY was 13.1 and 22.4 Mg Ha^−1^ for Est and Heelsum, respectively. Likewise, average TN was 136 tubers in Est and 199 tubers in Heelsum. Trial means for TV were comparatively more stable between trial locations with means of 21.3 cm^3^ in Est and 24.2 cm^3^ in Heelsum. Along with differences in mean hybrid performance, there was also greater dispersion of phenotypes in Heelsum than in Est. This was especially apparent for TY in Heelsum which displayed a standard error twice the size of TY in Est. This same marked difference could also be seen in TN where BLUPs in Heelsum exhibited a standard error 1.6 times greater than that which was found in Est. TV in contrast to the other phenotypes exhibited similar BLUP distributions between both trial locations. Examining these BLUP’s in light of each trait’s variance components (see Supplementary Table 3) suggests that TV showed the greatest stability of all 3 phenotypes.

### Variance ratios

Variance estimates were derived for all specified random effects for models *M*_0_ and *M_f_*. TV not only exhibited the largest proportion of variance explained by SCA ($d^{2}=0.14$) but also had the largest total genetic variance of any trait ($H^{2}=0.86$) (Fig. 2). TN and TY harbored similar proportion’s of SCA ($d^{2}=0.12$) with a considerable portion of nonadditive effects being partitioned in the SCA by environment interaction (Fig. 4). Broad-sense heritabilities were quite similar between TN (0.74) and TY (0.71) with the primary difference between the 2 traits being the partitioning of genotype by environment interactions ($\frac{\sigma_{ae}^{2}}{\sigma_{GE}^{2}}$ equal to 0.78 in TY in contrast to 0.65 in TN) and size of the residual variance (Fig. 4). The additive genetic component was the largest genetic effect across all traits with the ratio of additive genetic variance being identical in TY and TN (0.84) and nearly identical in TV (0.83). Between models *M*_0_ and *M_f_*, partitioning of variance changes most drastically for $\sigma_{ae}^{2}$. These were much larger in *M_f_* in TN and TY with the incorporation of the SCA main effect and SCA by environment interaction (Fig. 4).

### Genetic correlations

Along with within-trait variance ratios, genetic correlations were also computed using covariances extracted from model *M_f_*. These were produced for GCA (Fig. 5) and SCA (Fig. 6) and are shown together with their BLUP distribution for reference. The GCA intraclass correlation coefficient was found to be highest in TV (0.84) followed by TN (0.76) and TY (0.64) (Fig. 5). Also noteworthy, large within-trial GCA genetic correlations were found between TY and each of its yield components, TN (0.80) and TV (0.64) according to expectation. Little to no genetic correlation could be found between TN and TV (0.06). There were minor discrepancies when comparing each of these with their between-trial genetic correlations counterparts ($\rho_{TY,TN}=0.81,\rho_{TY,TV}=0.65,\rho_{TN,TV}=0.11$).

Similar multivariate trends were observed for SCA genetic correlations, though, with globally smaller values. The SCA intraclass correlation was highest in TV (0.75) with little difference between TY (0.55) and TN (0.53) (Fig. 6). Relatively large within-trial genetic correlations were observed between TY and TN ($\rho_{TY,TN}=0.73$) and between TY and TV ($\rho_{TY,TV}=0.69$). Genetic correlations between TN and TV were virtually null ($\rho_{TN,TV}=0.03$) showing little to no covariance between these 2 traits for SCA. Examining between-trait between-trial correlations only had minor deviations with respect to within-trial genetic correlations between TY and TN ($\rho_{TY,TN}=0.76$) and TN and TV ($\rho_{TN,TV}=-0.01$); however, these correlations between TY and TV were distinctly smaller than their within-trial counterparts ($\rho_{TY,TV}=0.55$) (Fig. 6).

**Fig. 7.**
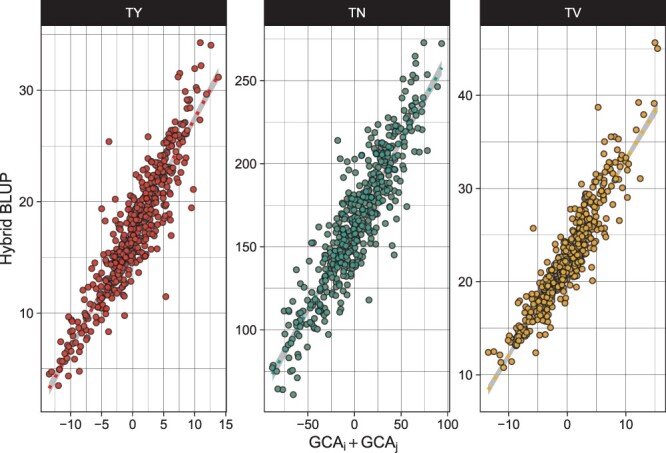
Scatter plot of hybrid BLUPs regressed on the GCAs of parents’ *i* and *j* for TY (Mg Ha^−1^), TN (total number of tubers per plot), and average TV (cm^3^).

When comparing the GCA and SCA quantiles for each trait and trial location, the GCA BLUPs were consistently larger than those SCA BLUPs. On average, any given GCA quantile was 2 times larger than its respective SCA quantile; this was true for all traits measured here (see Supplementary Table 4). These differences in magnitude between the estimated GCA and SCA effects could also be readily seen while examining the size of each variance component (Fig. 4) or even through a simple regression of hybrid BLUPs on the mid-parent value (Fig. 7). No linear relationship could be found between the estimated GCA and SCA effects for any trait (see Supplementary Fig. 1) coinciding with our model assumptions in (5).

### Model testing

The likelihood ratio tests conducted between model *M_f_* and *M*_0_ were found to be significant yielding a probability less than 0.001 (Table 3). This testing procedure was also applied on the univariate equivalent of these models for each trait and were all also found to be significant. Each test conformed with the AICs of each model pair where the smallest AIC was observed in the full genetic model suggesting that the best fit was achieved with the inclusion of SCA and its environmental interaction. This at the very least lends a statistical justification for these nonadditive genetic effects in all 3 tuber traits.

## Discussion

### Presence of additive vs nonadditive effects

SCA was detected across all phenotypes (Table 3) warranting sufficient evidence that SCA can impact yield, especially in its simpler components, seen primarily in TV (Fig. 4). However, the magnitude of GCA effects was far greater than the magnitude of SCAs estimated across all traits irrespective of their heritability or size of SCA variance. The GCA quantiles were between 1.4 and 2.5 times larger than their respective SCA quantiles (see Supplementary Table 4) suggesting a systematic importance of the additive genetic effects in this DHP population. The implications then are that most of the variation in the progeny can be found through the additive genetic variation in the parents. This was most apparent for TV ($h^{2}=0.71$) where regression of hybrid performance on the combination of parental GCAs had the best fit of all traits (Fig. 7). This illustrates that the parental GCAs (or, identically, the mid-parent value) capture the majority of a hybrid’s phenotype.

While this study is the largest of its kind, it is certainly not alone in attempting to decompose genetic components of yield in potato. A number of similar populations were used in both diploid and tetraploid backgrounds with wide-ranging results. Many of these studies came to find little nonadditive genetic variation for yield components similar to the results presented here with GCA being the primary component for traits like average tuber weight, tuber shape, total TN, and TY (Veilleux and Lauer 1981; Brown and Caligari 1989; Neele *et al.* 1991). These studies utilized either variants of a diallel or factorial crossing schema making the structure of their statistical models not altogether different than the modeling endeavored here. One exception between models’ (4) and (5) and those used in the aforementioned tetraploid populations is that variance attributed to GCA has a different interpretation due to differences in ploidy and levels of inbreeding. This said there is a large body of work that also finds SCA to be the largest (and at times, the only) detected effect in several complex traits. The same previously mentioned traits as well as others like incidence of hollow heart and tuber uniformity were also found to be predominately under the control of SCA (Killick 1977; Veilleux and Lauer 1981; Thompson and Mendoza 1984; Haynes 2001). Most notably, Tai (1976) was only able to detect SCA in their partial diallel crosses with no GCA component found for marketable yield and marketable TN.

The lack of an empirical consensus on the predominance of GCA and SCA in potato is not very surprising in of itself. The estimation of these parameters is very much contingent on numerous factors including crop ploidy, genetic background, number of parents, degree of environmental stress, and choice of statistical model (to name a few), all of which are prone to change across experiments. Even making comparisons between studies utilizing very similar genetic backgrounds can lead to divergent findings (Tarn and Tai 1977; Maris 1989). While seemingly incoherent, the following does offer some interesting grounds for considering those genetic effects observed here. Many of the aforementioned studies estimated variance components on populations that had undergone significant selection through a recurrent selection schema (Maris 1989; Haynes 2001) or were themselves the product of strong selection on GCAs in their ancestors (Tai 1976). In both cases, less additive genetic variation can be expected among hybrids derived from them leaving nonadditive genetic variation to be the predominant genetic effect within their interpopulation crosses. Conversely, populations like ours which show ample additive genetic variation might be younger with respect to selection pressure in their ancestors on these traits; though without any formal analysis on population structure this is speculative. Another line of reasoning for the smaller SCAs found here relative to many of the tetraploid studies could be explained by *progressive heterosis* whereby higher-order nonadditive genetic effects become possible through polyploidy (Birchler *et al.* 2003). Recent genomic studies in tetraploid potato support this hypothesis with evidence of a genetic residual effect (which could be explained by tri and quadrigenic dominance) contributing as much as 45% of total genetic variation in potato yield (Endelman *et al.* 2018). However, making any meaningful confirmation on the specific role of ploidy in producing nonadditive genetic effects is beyond the scope of this present study and only deserves a cursory mention.

### Genetic architecture of yield

Among our 2 yield components (i.e. TN and average TV), we found strong genetic correlations between each and TY for both the additive (see Fig. 5) and nonadditive genetic effects (see Fig. 6). Numerous studies have identified these same phenotypes as major determinants of total tuber yield marking them both as strategic heritable targets for breeding (Thompson and Mendoza 1984; Khayatnezhad *et al.* 2011). Consistent with these studies, TN GCAs appeared to impact TY more than TV with $\rho_{TN,TY}$ equaling 0.80 and a $\rho_{TV,TY}$ of 0.64. SCA genetic correlations behaved similarly with $\rho_{TN,TY}$ equal to 0.73 and $\rho_{TV,TY}$ equal to 0.69. Interestingly, while TV had the greatest stability with respect to both GCA (*ICC_gca_* = 0.84) and SCA (*ICC_sca_* = 0.75), the between-trial genetic correlations in $\rho_{TV,TY}$ dropped to 0.55 suggesting less coupling in SCA by trial response with TY in contrast to the between-trial genetic correlations seen in TY and TN ($\rho_{TN,TY}$=0.76). These additive and nonadditive components point to TN being the primary determinant of yield in this hybrid population.

Among certain market classes, our 2 yield components, average TV (or tuber size) and TN, often exhibit an inverted relationship due to the physiological and genetic limits of potato. For example, Thompson and Mendoza (1984) found a genetic correlation of −0.24 among their panel. Additionally, Lemaga and Caesar (1990) identified negative cubic trends between TN and average tuber weight capturing a majority of variation. Interestingly, no meaningful relationship could be found between these 2 yield components with respect to additive (Fig. 5) and nonadditive genetic correlations (Fig. 6). To repeat our previous suspicion, this suggests a lack of directional selection on one of these 2 traits evident by the lack of genetic constraints between them (Blows and Walsh 2009). TV had the largest proportion of additive variation (Fig. 4) and genetic variation in general (Table 2) suggesting little to no direct selection on this component of yield. These properties then make this population an interesting candidate for future selection given its genetic potential to be adapted to a variety of tuber types. Another oddity to consider here is that while SCA was detected independently in these 2 yield components, this did not manifest in the increase of SCA in TY, but very much the opposite. Others have identified that heterosis in these same yield components was responsible for a geometric increase in gross yield (Tarn and Tai 1977). Further multivariate studies of vigor in diploid potato could further elucidate SCA architecture especially as selection pressure is applied, a key scenario within breeding programs.

### Using GCA and SCA in commercial breeding

The large narrow-sense heritabilities and magnitude of the GCAs found here have major implications for breeders of DHP. To begin, the valid estimation of GCAs further validates the potential of potato in its conversion into an inbred–hybrid crop as purported before (Lindhout *et al.* 2011; Jansky *et al.* 2016). Furthermore, the size of the estimated GCAs relative to a hybrid’s average performance (Fig. 7) shows that standard breeding designs used in other hybrid crops will likely be just as efficacious in DHP. For example, the use of test crosses, a mainstay in maize breeding, can also be utilized in evaluating the performance of potato parental lines for hybrid crosses with reasonable success. These test crosses can be further utilized for model training and be the basis for genomic selection of parents (using breeding values) or hybrid crosses (via mid-parent value); again, a standard-place method in hybrid breeding (Albrecht *et al.* 2011). To quickly add, while we found little contributions made by SCA, their relevance to breeders does not necessarily end here. Depending on the specific mechanism behind these observed nonadditive effects, they could be further exploited in the trait of interest through initial breeding design (e.g. formation of heterotic pools). Heterotic breeding has become a major target for quality trait improvement in other solanaceous crops including chilli pepper (Herath *et al.* 2020), eggplant (Kumar *et al.* 2020), and tomato (Frankel 1983). This is where potato meets an interesting intersection between the vegetable and agronomic worlds where SCA might play a more valuable role for qualities controlling specific market criteria (e.g. average TV, tuber length, and shape) but would be less emphasized in composite and complex traits (e.g. gross yield, starch, and protein content) where GCA is the predominant genetic effect at play. Having said this, future work into the biometric mechanism of vigor will be able to lend more wisdom for how breeders should wield this in a hybrid potato breeding program.

## Conclusion

### Limitations

The application of these results should be done with some qualification. One principal limitation of this study can be found in the number of trials conducted. All inference drawn here was based upon only 2 trials that took place over one season. This limits our findings to one particular year which narrows their interpretive weight and scope. Related to this, this study was performed on a particular composite experimental population and does not necessarily represent the heterotic potential of the entire tuber-bearing Solanum gene pool. Considering all this, this population is an appropriate candidate for the purpose of surveying the presence and potential of heterotic vigor in DHP which was the aim of this current paper.

### Future work

Finding evidence for heterotic effects in DHP does not yield much regarding the source of the effects identified here. The statistical models assume all underlying effects captured by the SCA term to be the product of cumulative dominance deviations across the genome, but there exist many other plausible sources of nonadditive variation. Since its conception, genetic theory has explained heterosis with a whole suite of models with many being broadly plausible (see Labroo *et al.* 2021). However, these apparent nonadditive effects could just as simply be explained by dispersion of additive alleles among parents, a hypothesis that is generally supported empirically (Frankel 1983; Mackay *et al.* 2021). Nevertheless, these effects continue to be interesting point of study and still remain an important target in hybrid breeding of modern crops. This is especially worthwhile in DHP given its novelty as a hybrid crop with the potential of heterotic breeding to still be determined.

This is the first study in DHP to produce estimates of general and specific combining abilities using a large panel of commercially derived parents. This represents a major milestone in the reorientation of potato from a clonal tetraploid to a diploid inbred–hybrid crop. Identifying the predominance of additive genetic effects for multiple yield components among hybrids offers strategic insight on the necessity of effective generation of parental lines and early population development in general. Though the estimated nonadditive effects in this population are smaller in contrast to their additive counterparts, heterotic vigor shows some minor role in simpler traits. Specific quantitative traits should be targeted for SCA exploitation to bolster variety development on top of their parental effects. Further research into the genetic mechanisms for the apparent nonadditive effects will also better elucidate the strategic advantage (if any exist) in key economic targets in hybrid potato.

## Data availability

All trial and pedigree data utilized for the following analysis have been made available on GSA figshare (https://doi.org/10.25387/g3.16973293). File Phenotypes.csv contains the 3 aforementioned traits along with field trial row, column, and block indices for each observation. File Pedigrees.csv gives a hybrid identification number with each parental code.

## Funding

The research produced for this publication was made possible through funding provided by Topsector Tuinbouw & Uitgangsmaterialen in the “Re-booting potato: enhancing the breeding of hybrid diploid potato” project (project number: TU-1855).

## Conflicts of interest

All authors of this publication have reviewed the topic of any potential conflicts of interest and have concluded that there are none relevant to the research discussed in this article. We feel that our original statement expressed this sentiment concisely.
